# Ignored Role of Paroxysmal Atrial Fibrillation in the Pathophysiology of Cryptogenic Stroke in Patients with Patent Foramen Ovale and Atrial Septal Aneurysm

**DOI:** 10.2174/011573403X267669240125041203

**Published:** 2024-02-01

**Authors:** Ertan Yetkin, Hasan Atmaca, Bilal Cuglan, Kenan Yalta

**Affiliations:** 1 Division of Cardiology, Türkiye Hospital, Istanbul, Turkey;; 2 Department of Cardiology, Kanuni Sultan Suleiman Training and Research Hospital, Istanbul, Turkey;; 3 Department of Cardiology, Faculty of Medicine Edirne, Trakya University, Trakya, Turkey

**Keywords:** Stroke, cryptogenic stroke, atrial septal aneurysm, patent foramen ovale, atrial fibrillation, pathophysiology

## Abstract

The association between cryptogenic stroke (CS) and patent foramen ovale (PFO) with or without atrial septal aneurysm (ASA) has been a debate for decades in terms of pathophysiologic processes and clinical courses. This issue has become more interesting and complex, because of the concerns associating the CS with so-called normal variant pathologies of interatrial septum, namely ASA and PFO. While there is an anatomical pathology in the interatrial septum, namely PFO and ASA, the embolic source of stroke is not clearly defined. Moreover, in patients with PFO and CS, the risk of recurrent stroke has also been associated with other PFO-unrelated factors, such as hyperlipidemia, body mass index, diabetes mellitus, and hypertension, leading to the difficulty in understanding the pathophysiologic mechanism of CS in patients with PFO and/or ASA. Theoretically, the embolic source of cryptogenic stroke in which PFO and/or ASA has been involved can be categorized into three different anatomical locations, namely PFO tissue and/or ASA tissue itself, right or left atrial chambers, and venous vascular territory distal to the right atrium, *i.e.*, inferior vena cava and lower extremity venous system. However, the possible role of paroxysmal atrial fibrillation associated with PFO and/or ASA as a source of cryptogenic stroke has never been mentioned clearly in the literature. This review aims to explain the association of cryptogenic stroke with PFO and/or ASA in a comprehensive manner, including anatomical, clinical, and mechanistic aspects.

The potential role of paroxysmal atrial fibrillation and its contribution to clinical course have been also discussed in a hypothetical manner to elucidate the pathophysiology of CS and support further treatment modalities.

## 1 INTRODUCTION

The association between cryptogenic stroke (CS) and patent foramen ovale (PFO) with or without atrial septal aneurysm (ASA) has been a debate for decades in terms of pathophysiologic processes and clinical courses. This issue has become more interesting and complex, because of the concerns associating the CS with so-called normal variant pathologies of interatrial septum, namely ASA and PFO. Cryptogenic stroke comprises up to 40% of ischemic stroke not only in the adult population, but also in adolescents and children [1-4].

The prevalence of PFO has been reported up to 6 times higher in patients with CS being less than 55 years of age compared to those with stroke having known etiology [5]. Although anatomic pathology exists at the interatrial septum contributing to CS, the origin of emboli has not been clearly described.

This review aims to explain the association of cryptogenic stroke with PFO and/or ASA in a comprehensive manner, including anatomical, clinical, and mechanistic aspects, mainly focusing on the presence of paroxysmal atrial fibrillation (PAF). The potential role of paroxysmal atrial fibrillation and its contribution to clinical course have been also discussed in a hypothetical manner to elucidate the pathophysiology of CS and to support further treatment modalities.

## 2 PATENT FORAMEN OVALE AND ATRIAL SEPTAL ANEURYSM

The foramen ovale, which is an obligatory hole of intrauterine life to maintain normal fetal circulation, usually closes after birth. When this closure does not take place or the foramen ovale fails to close, it is called PFO. The prevalence of PFO has been reported in up to 25% of individuals in autopsy and transesophageal echocardiography studies [6, 7].

Embryologically, the formation of right and left atrium is accomplished by the formation of interatrial septum through a sequential formation of septum primum, ostium primum, ostium secundum, septum secundum, and foramen ovale in a physiologically oriented manner. As the septum primum grows towards the endocardial cushion from the posterior roof of the common atrium, forming ostium primum, continued fusion of the endocardial cushions closes the ostium premium. Meanwhile, fusion of the small fenestrations at the upper part of the septum primum forms the ostium secundum. Thereafter, ostium secundum is closed over by another structure called septum secundum, eventually forming foramen ovale. During fetal life, the caudal portion of the septum primum functions as a valve of foramen ovale, allowing blood flow from the inferior vena cava into the left atrium and systemic circulation of the fetus. After birth, starting from the first breath, the valve closes the foramen ovale. This functional closure of the foramen ovale is followed by anatomical closure. However, the closure fails in up to 25% of individuals, resulting in blood flow through the foramen ovale between the edges of the septum secundum and septum primum into the left atrium [4, 6, 8]. Another pathology of the interatrial septum, the atrial septal aneurysm is a congenital deformity at the region of fossa ovalis, in which septum primum forms the floor and is surrounded by the limbs of septum primum and secundum [2-4]. ASA is characterized by redundant mobile and thin interatrial septal tissue bulging into the left or right atrium, although differences between the interatrial pressure have been reported as a cause of its development, ASA is known as a congenital abnormality of septum primum at the region of fossa ovalis or entire septum [9, 10]. ASA has been reported to co-exist with PFO in 60% to 80% of the cases and has a prevalence rate of 2-3% in the general population [11]. In addition to the significant association between PFO and ASA, both pathologies have been reported to be independent predictors of embolic events in patients with normal carotid arteries. Additionally, more than two-thirds of the patients with ASA also have PFO [12]. A recent meta-analysis has revealed that PFO patients with ASA carry a higher risk of CS compared to those without ASA, indicating additional contribution of ASA to CS [13].

Although both the PFO and ASA are hemodynamically silent and innocent cardiac abnormalities, they have been associated with a variety of diseases and pathologies, such as migraine, stroke, transient global amnesia, platypnea-orthodeoxia syndrome, obstructive sleep apnea syndrome, aortic aneurysm, mitral and aortic valvular regurgitation [9,14-17].

## 3 PATHOPHYSIOLOGY OF CS IN PFO AND ASA

**Fig. (1):**
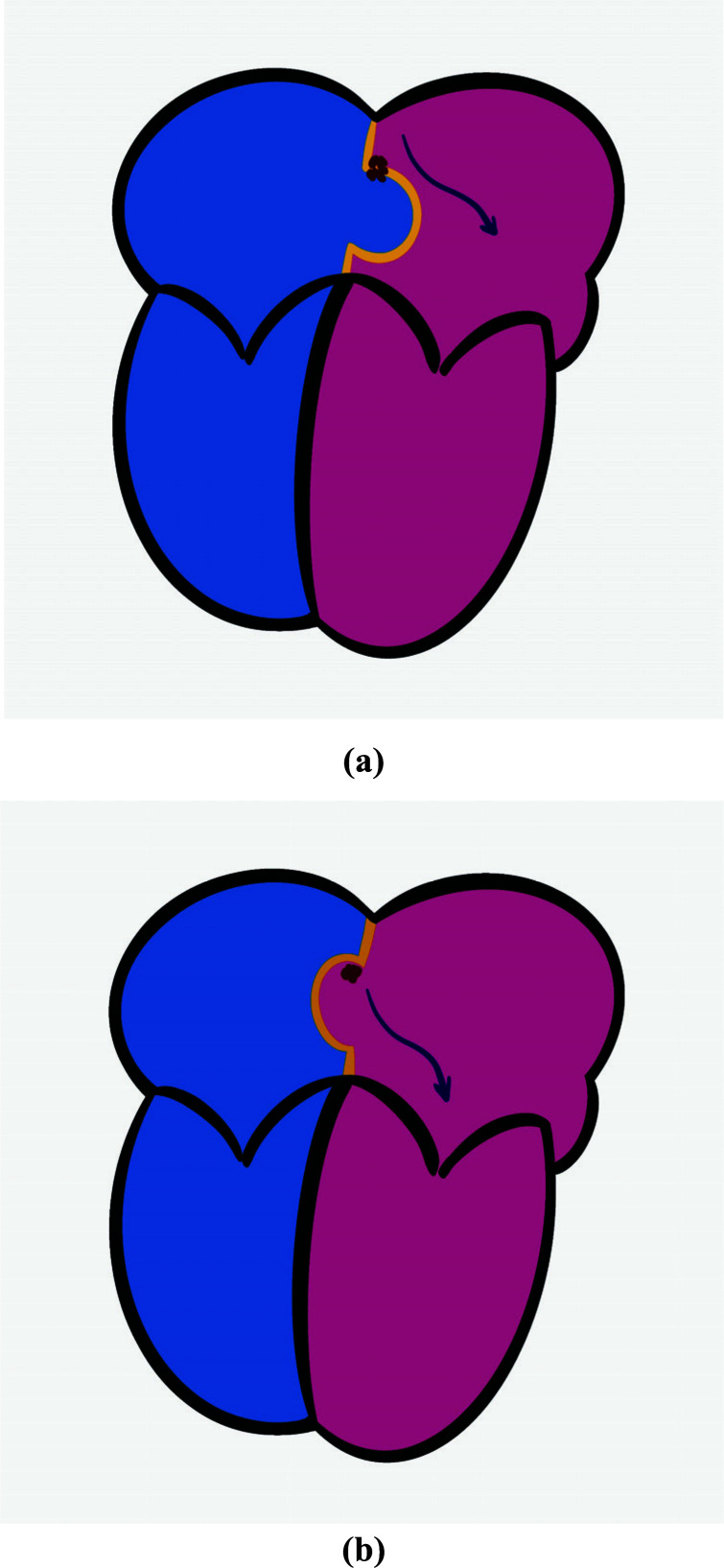
Schematic illustration of the mechanism of cryptogenic stroke in patients with patent foramen ovale and atrial septal aneurysm. Dark red dots show *in situ* thrombus formation at the site of patent foramen ovale (**a**) and aneurismal recess (**b**).

Theoretically, embolic sources of cryptogenic stroke in which PFO and/or ASA are involved can be categorized into three different anatomical locations. These locations are [1] PFO tissue and/or ASA tissue itself [2], right or left atrial chambers [3], venous vascular territory distal to the right atrium, *i.e.*, inferior vena cava, and lower extremity venous system. Hypothetically, *in situ* thrombus formation has been supposed to be occurring at the tunnel of PFO or the recess of aneurismal tissue (Figs. **1a** and **1b**) [1, 5, 18]. Stasis of blood flow due to the tunnel-like structures of PFO or in the recess of aneurismal atrial septal structure or flaps facilitates the formation of a thrombus. In the case of ASA, the formation of a thrombus at the left atrial side of the aneurysm and subsequent dislodgement during the oscillation of the aneurysm are likely explanations for *in situ* thrombus formation as the potential mechanism of CS.

**Fig. (2):**
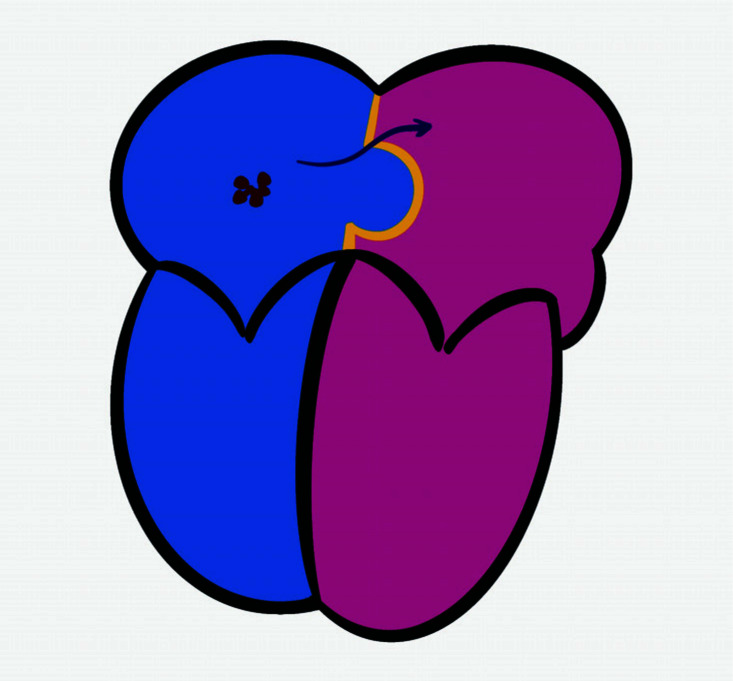
Schematic illustration of the mechanism of cryptogenic stroke in patients with patent foramen ovale and atrial septal aneurysm. Dark red dots show the remote embolic source in the right atrium and the arrow shows the route of paradoxical embolism.

In the scenario of paradoxical embolisation through PFO, the existence and dislodgement of remote thrombus are obligatory (Fig. **2**). The most likely sources of thrombus are the right atrium and the venous vascular territory flowing through the inferior and superior vena cava. Documented venous thrombus and PFO complicated by CS have been reported sparsely in the literature [19-24].

Along with the paradoxical embolism, the thrombus crossing through the PFO, the so-called “thrombus in transit”, has been visualized in a large number of case reports (Fig. **1a**). However, these cases are mostly complicated by pulmonary embolism, in which increased right atrial pressure and increased thrombus mass might facilitate the transition of thrombus through the PFO [21, 23, 24].

Recent studies have shown significant associations of ASA with structural (valvular regurgitation, ascending aortic dilatation) heart diseases and rhythm disturbances (premature atrial and ventricular contractions, supraventricular tachycardia, and atrial fibrillation) [12, 25, 26]. The occurrence of thrombus in the left or right cavity itself and subsequent embolisation are other mechanisms of CS. In this regard, arrhythmic associates of ASA, in particular AF, might play a role as an underlying reason for CS. Although the literature lacks definite evidence, this issue has been mentioned in a recent critical review by Leonard *et al*. [5]. On the other hand, evidences exist that support the triggering role of AF for CS in patients with ASA [5, 25, 27]. Electrophysiologic changes, such as p-wave dispersion and atrial electromechanical delay, which have been documented in patients with ASA, are likely to play a substrate role in the occurrence of PAF and supraventricular tachycardia [25, 28, 29]. Likewise, an increased prevalence of AF and supraventricular tachycardia has been reported in patients with ASA [25, 28]. Moreover, ASA has been found in 8% of patients with cerebral embolism of unknown origin [29].

**Fig. (3):**
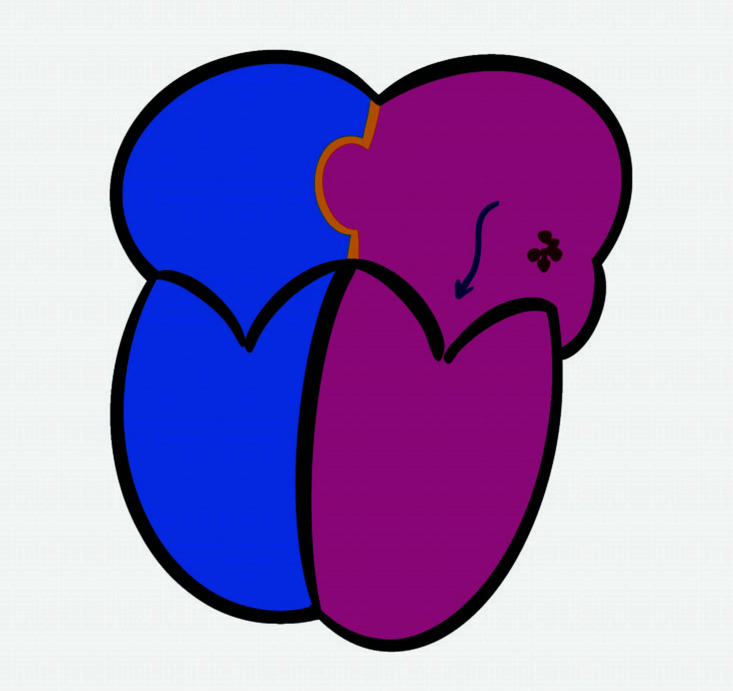
Schematic illustration of the mechanism of cryptogenic stroke in patients with patent foramen ovale and atrial septal aneurysm. Dark red dots show the embolic source in the left atrium possibly due to paroxysmal atrial fibrillation.

Paroxysmal AF-induced formation of thrombus in the left atrium and subsequent embolisation rather than the *in situ* thrombus formation in the tunnel of PFO and recess of ASA seem to be a more reasonable explanation for the occurrence of CS (Fig. **3**). In this regard, the non-superiority of PFO closure to medical treatment for preventing the recurrence of cerebral thromboembolic events [30-32] underlines the idea that PFO is not the only case directly related to CS. A recent analysis has revealed that the presence of an ASA, rather than shunt size, is the major contributing factor to the occurrence of recurrent stroke in patients with PFO [33]. Moreover, the presence of ASA has also been related to hospital recurrence of cryptogenic stroke [1]. In this regard, AF should be taken into consideration in the discussion about the pathophysiological mechanisms of CS in the presence of PFO and ASA. Indeed, attention has already been paid to patients with CS. Continuous electrocardiographic monitoring using an implantable loop recorder (ILR) has been shown to detect the presence of PAF or so-called subclinical AF in up to 30% of patients with CS in the general population [34-36]. Continuous electrocardiographic monitoring by ILR has resulted in a three times increase in the detection of PAF in patients without stroke, but with a risk of stroke [37]. In this regard, continuous electrocardiographic monitoring might be a reasonable method to focus on the presence of AF attacks or so-called asymptomatic AF episodes in patients with PFO and/or ASA. A recent meta-analysis has revealed that ASA confers an additional risk of stroke in the presence of PFO [38]. The question is whether the risk comes from the increased rate of paradoxical embolisation and *in situ* thrombus formation or the increased rate of ASA-associated PAF attacks. The presence of PAF in patients with PFO and/or ASA has never been evaluated in patients with CS.

## 4 PREVENTION OF CRYPTOGENIC STROKE AND PFO CLOSURE

The current strategies for the prevention of recurrent stroke in patients with CS and PFO and/or ASA include closure of PFO and medical therapy (antiplatelet or anticoagulant therapy). The effectiveness of PFO closure to prevent recurrent stroke has been a debate due to controversial results in the literature. The heterogeneity of clinical studies (randomized controlled studies, observational studies) and differences in medical treatment regimens (anti-platelet treatment alone, anti-coagulant treatment, or both) are the potential contributing factors to this controversy.

Additionally, PFO-unrelated factors, such as hypertension, diabetes mellitus, and obesity, are likely to play a supplementary role in the pathophysiology of CS in patients with PFO and/or ASA. PFO may coexist with other potential risk factors in patients with CS, leading to difficulty in attributing the stroke to either the PFO or other PFO-unrelated factors. In CS patients with PFO, hypercholesterolemia and higher BMI have been found to be strongly related to prior stroke [39]. Likewise, diabetes mellitus, hypertension, and ischemic heart disease have been shown to be associated with the risk of CS in patients with PFO [40].

In the Gore REDUCE study, PFO closure plus anti-platelet therapy has been found to be superior to anti-platelet therapy alone for the prevention of recurrent stroke in patients with cryptogenic stroke attributable to PFO [11]. In the CLOSURE-1 trial, PFO closure alone has not been found to be superior to anti-platelet therapy alone [30]. Among patients having recent cryptogenic stroke attributed to PFO with an associated ASA and large inter-atrial shunt, PFO closure plus anti/platelet therapy has resulted in a lower rate of recurrent stroke compared to those given anti/platelet therapy alone [10]. There are several subtle aspects that should also be discussed regarding the contradictive results of RCTs [10, 11, 30, 31, 39-41] and meta-analysis [42-44].

A recent meta-analysis, including both RCTs and observational studies, has reported PFO closure to significantly reduce the risk of recurrent ischemic neurological events compared to anti-platelet therapy alone. However, the same analysis has also underlined the non-superiority of PFO closure when compared to the mixed medical therapy group, *i.e.*, anti-platelet and anticoagulant treatment [42]. Pan *et al*. paid more attention to anticoagulant treatment while discussing the individual assessment of patients and tailoring the medical therapy [42]. It is of note that the possible co-existence of PAF in patients with PFO and/or ASA might be discussed in the pathophysiologic aspects of CS. Unfortunately, PAF has never been mentioned and investigated neither in RCTs nor in meta-analyses. There are several rationales to discuss the role of PAF in the scenario of CS in patients with PFO and ASA, firstly the ineffectiveness or controversial results regarding PFO closure for CS. Secondly, adding anticoagulant therapy to the medical treatment arm has made the ineffectiveness of PFO closure more profound. Regarding the independent association of PFO and ASA with rhythm disturbances, *i.e.*, AF, we may assume that the role of AF in the pathogenesis of CS has been overlooked, while there has been a focus on PFO and ASA. The arrhythmic event might serve as the pathophysiological trigger of stroke in patients with ASA and PFO, where the anatomical structure appears insufficient to justify the pathogenesis of cryptogenic stroke in the absence of other risk factors. Although *in situ* thrombosis or paradoxical embolism can explain the pathophysiology of stroke to some extent in patients with PFA and ASA, the literature still lacks definitive evidence regarding the cause of CS, except for the anatomical existence of PFO and ASA. The gray area in the pathophysiology of CS is where the thrombus occurs. *In situ* thromboses and embolic sources from the right atrium are the conventional but weak pathophysiological explanations for CS. However, it is possible that emboli might have arisen from the left atrium itself. In this regard, the contribution of PAF to CS would be a reasonable explanation in patients with PFO and ASA. Therefore, focusing on the left side of the atrium rather than PFO solely would improve our understanding of the dilemma of CS and would enable us to introduce new therapeutic approaches. Within this context, documentation of PAF by rhythm Holter recording or event loop recorders has paramount importance in the clinical course of CS in patients with PFO and/or ASA [45].

## CONCLUSION

The existence of paroxysmal AF should be assessed more rigorously in daily clinical practice using continuous cardiac monitoring systems, and the clinical relevance of PAF with PFO and/or ASA in CS should be elucidated in future randomized clinical studies. Thereafter, in addition to PFO closure, administration of oral anticoagulant would provide a further reduction in the recurrence rate of CS. In order to prevent the possible occurrence of PAF attacks, the inclusion of anti-arrhythmic agents, *i.e.*, beta-blockers, for selected patients might be a reasonable option against the recurrence of CS. Therefore, further prospective clinical studies are warranted to evaluate the effects of anti-arrhythmic treatment on the prevention of CS.

## LIST OF ABBREVIATIONS

ASA: Atrial Septal Aneurysm
CS: Cryptogenic Stroke
PFO: Patent Foramen Ovale
PAF: Paroxysmal Atrial Fibrillation
